# Chronically ill children’s participation and health outcomes in shared decision-making: a scoping review

**DOI:** 10.1007/s00431-021-04055-6

**Published:** 2021-04-05

**Authors:** R. O. Wijngaarde, I. Hein, J. Daams, J. B. Van Goudoever, D. T. Ubbink

**Affiliations:** 1 Amsterdam UMC, University of Amsterdam, Emma Children’s Hospital, Room H8-247, Meibergdreef 9, 1105 AZ Amsterdam, The Netherlands; 2 Child and Adolescent Psychiatry and de Bascule, Academic Medical Center Amsterdam, Amsterdam, The Netherlands; 3 Medical Library, Amsterdam University Medical Centers, AMC, Amsterdam, The Netherlands; 4 Department of Surgery, Amsterdam University Medical Centers, AMC, Amsterdam, The Netherlands

**Keywords:** Shared decision-making, Decision aids, Self-determination, Patient-centered care, Triadic information exchange, Chronic illness

## Abstract

Based on the United Nations Conventions on the Rights of the Child (CRC), it is a child’s right to participate in all matters concerning its wellbeing. Little is known about chronically and/or critically ill children’s participation in pediatric shared decision-making (SDM). We explored medical literature to see if and how these children participate in pediatric SDM. We searched relevant medical databases published between January 2008 and January 2020 for studies targeting children aged 4–18 years old, suffering from a chronic and/or critical disease. We found 9 relevant studies. SDM interventions mostly used were decision aids (*n*=8), questionnaires for caretakers/parents and children (*n*=4), and a SDM toolkit (*n*=2). Perceived involvement in SDM and knowledge increased amongst children, adolescents, and caretakers following these interventions. Decisional conflict measured using the 0–100 point DCS scale (higher scores indicate more decisional conflict) was reduced by 15.9 points in one study (*p*<0.01) and 17.8 points in another (95%CI: 13.3–22.9). Lower scores were associated with higher satisfaction with the decision aid by children, caretakers, and clinicians.

*Conclusion*: Stakeholders should advocate initiatives to facilitate a child’s participation preferences regarding pediatric SDM since decision support tools help chronically ill children to be more involved in SDM as they increase the children’s knowledge and satisfaction and reduce decisional conflicts.

**Table:** 

**What is Known:** *• Decision aids can help improve participation, knowledge, satisfaction, and health outcomes.* *• Quality and consistency of the information exchange impact quality and outcome of SDM*.	
**What is New:** *• Depending on a child’s age, evolving capacities, and communication and participation preferences, more evidence is needed on which tools are suitable for chronically ill children to ensure their preferred participation in pediatric SDM.* *• Pediatricians adopt healthcare SDM tools and techniques that do not always take into account that a child’s right to participate in pediatric SDM including the tendency to use interventions that are not specifically designed for pediatrics.*

**What is Known:**

*• Decision aids can help improve participation, knowledge, satisfaction, and health outcomes.*

*• Quality and consistency of the information exchange impact quality and outcome of SDM*.

**What is New:**

*• Depending on a child’s age, evolving capacities, and communication and participation preferences, more evidence is needed on which tools are suitable for chronically ill children to ensure their preferred participation in pediatric SDM.*

*• Pediatricians adopt healthcare SDM tools and techniques that do not always take into account that a child’s right to participate in pediatric SDM including the tendency to use interventions that are not specifically designed for pediatrics.*

## Introduction

Based on the United Conventions on the Rights of the Child, it is a child’s human right to participate in all matters concerning its wellbeing. Shared decision-making (SDM) is a multi-step process that involves relationship-building between clinician and patient with the aim of sharing information, such as the patient values, recommended treatment options, and the risks and benefits of these options. Thus, after deliberation with the clinician, patients can express their preferred (non)treatment choice based on clinicians’ advice, available evidence, and their own personal views and beliefs [1–4]. Ongoing national initiatives promote the use of SDM in pediatric clinical practice [5–9]. A systematic review summarized the effect of SDM inventions in pediatrics on patient-centered outcomes and indicated that the use of SDM interventions in pediatrics increases patient knowledge and decreases decisional conflict [10]. However, sometimes SDM interventions in the pediatric field do not target children [10]. If they do, available studies show a lack of knowledge on how to optimize communication with the child and how to optimize child participation in decision-making according to his or her own preferences [11–13].

### Challenges in pediatric SDM

Several systematic reviews have addressed developments and deficits in pediatric SDM [10, 14–16]. These gaps in knowledge affect the quality and consistency of the information exchange and, consequently, the quality and outcome of a SDM process. Besides, it also affects the SDM goals such as quality and continuity of care and patient satisfaction [16, 17]. It is still largely unclear which interventions are effective and suitable for children, and which health outcomes can be attributed to SDM interventions [2, 3, 10, 14, 15, 18]. It is also unclear how communication with the child according to his or her own preferences should be optimized. A complicating factor in studying pediatric SDM is a certain lack of consensus about the most preferable definition of SDM [2, 3]. This lack of consensus about scope and meaning of SDM makes it sometimes difficult to determine which interventions, tools, and techniques amount to SDM and what health outcomes can be attributed to them. A consensus about which tools should be defined as such has recently been published [19].

### Shared decision-making, social vulnerability, and chronic diseases

Socially vulnerable groups of children could benefit in various ways from improved quality of the information exchange that underlies a pediatric SDM process [16]. This goes especially for children whom because of their ethnicity or demographic characteristics are disproportionally at risk of being affected by chronic diseases such as type 1 Diabetes, asthma, and sickle cell disease [20–22]. The aim of our study was to explore SDM interventions and their effectiveness in terms of participation, knowledge, decisional conflict, satisfaction health-related quality of life, and treatment adherence for chronically and critically ill children. These two groups of pediatric patients were selected because living with a chronic disease imposes various challenges for pediatric SDM such as a child’s evolving capacities, how to weigh his/her growing experience with living with a chronic condition, and the different possible dynamics and stages of the illness. For critically ill children, uncertainty regarding diagnosis and prognosis makes it harder to decide for all stakeholders which treatment option is in the child’s best interest.

## Methods

We conducted a scoping review, using the PRISMA guidelines and PRISMA-ScR Checklist by Tricco et al. as a reference [23] to analyze gaps in knowledge about health outcomes of child participation in pediatric SDM. We conducted a scoping rather than a full systematic review as we limited our search to identify key factors related to predefined health outcomes of child participation in pediatric SDM, rather than providing an overview of all available evidence on SDM tools in pediatrics. We believe that conducting this scoping review would give us better insight in pediatric SDM knowledge gaps. This would help us to narrow our focus to conduct a systematic review as a follow-up study.

### Data sources and search strategy

With the aid of a clinical librarian (JD) we designed a search strategy (Appendix) and conducted literature searches in MEDLINE, PsycInfo, Communication and Mass Media Complete, and Web Of Science, between January 2008 and January 2020. We included both observational and experimental study designs that addressed child participation in treatment decision-making, knowledge, decisional conflict, health-related quality of life, and treatment adherence. Abstracts or letters were excluded. Studies that did not report of the predefined health outcomes of child participation in SDM were also excluded. Screening the studies for eligibility was performed by two co-authors (ROW, IH) independently. Reference lists were checked for relevant studies. No language restrictions were applied.

### Study selection

We uploaded the resulting dataset in the Rayyan software [24], to independently assess the eligibility of the studies found. Reviewer 1 (ROW) first screened the identified titles and abstracts to determine their relevance for this study. Reviewers 1 and 2 (IH) independently scanned the studies for eligibility based on our inclusion and exclusion criteria. After initial screening of 100 titles and abstracts, we calculated a Cohen’s Kappa (*κ*). If *κ* was ≥0.80, the first author would continue to screen the rest of the articles. If not, both authors would discuss their differences to reach a consensus. Any remaining discrepancies were solved by discussion.

### Data extraction and analysis

Reviewer 1 extracted data from the eligible studies using a predefined, custom-made data extraction form. Reviewer 2 double-checked the extracted data. Data extracted from child *participants* included the following: age, gender, illness, care setting, and treatment decision-making. Data from *interventions* comprised aims, content, technology, and online availability. Data from the *outcomes* included a child’s level of participation, number of participants, tools for participation, and the use of eHealth tools in pediatric SDM.

### Quality assessment, data analysis, and synthesis

The Cochrane handbook was used as a reference to assess the risk of selection, performance, detection, attrition, and reporting bias [20]. Data were presented as means or medians, whenever appropriate. Differences were expressed as risk differences (RD) and 95% confidence intervals (CI). Meta-analysis was planned if the children, SDM tool used, and outcome measure were similar. A random effects model would be used for pooling the data, if possible. As a measure of statistical heterogeneity, the *I*^2^ was used. If *I*^2^ was higher than 80%, pooling of data would be considered not meaningful and we would merely explore the outcomes.

## Results

### Characteristics of included studies

**Fig. 1:**
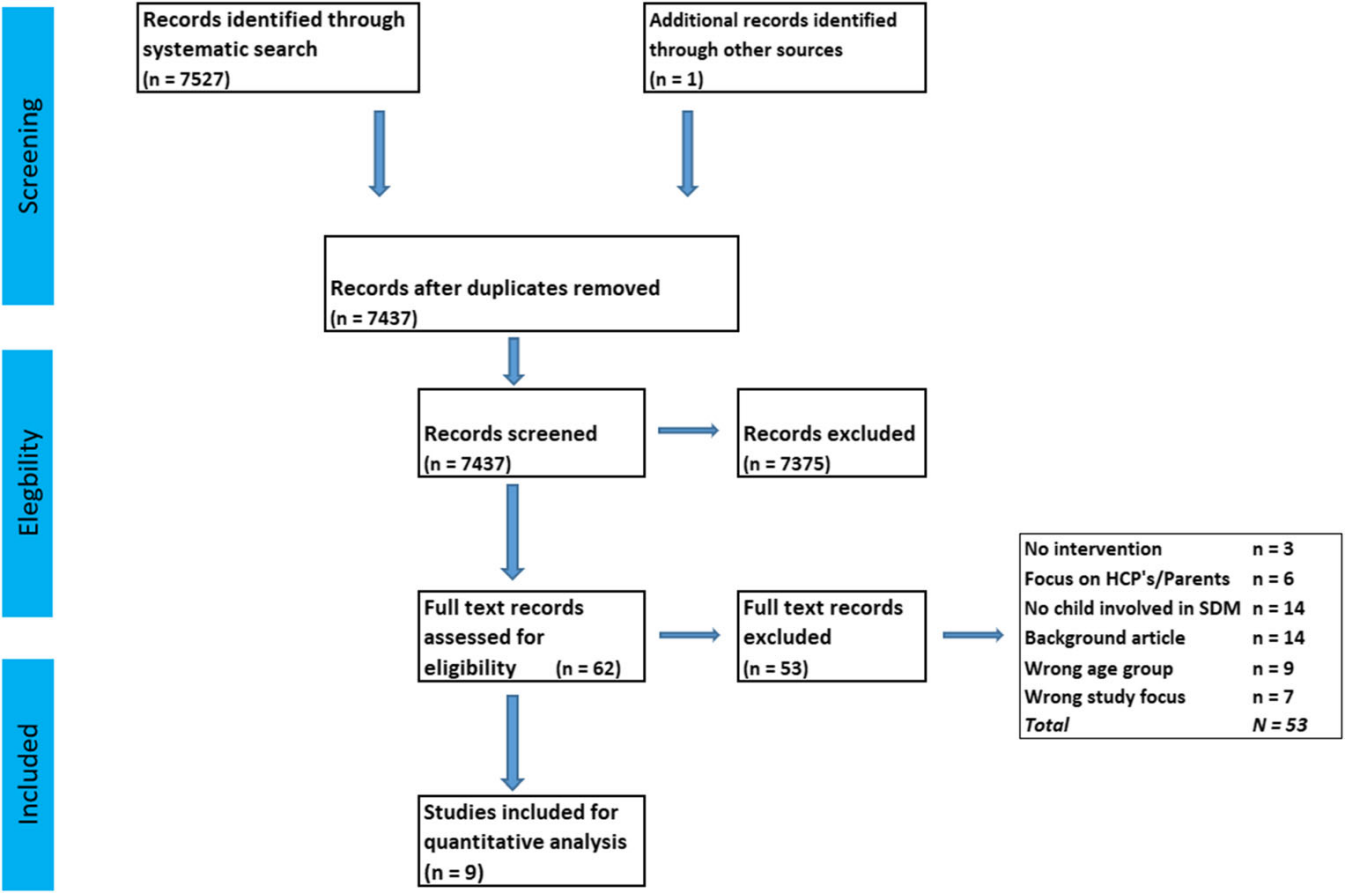
Search flowchart child participation in shared decision-making

The flow of study inclusion is shown in Fig. 1. After the second round of screening study eligibility, Cohen’s Kappa was 1.00. Hence, the first author continued to screen the remaining titles and abstracts. A total of 9 studies were included, comprising 2007 children. These were published between 2015 and 2020 and were conducted in the USA, Canada, Australia, and Egypt. Seven studies had a pre-post intervention design, and two were case series after an SDM intervention. Study sizes ranged from 11 to 746 children included. The children suffered from chronic disorders such as neuromuscular scoliosis, diabetes, asthma, juvenile inflammatory arthritis (JIA), obesity, and depression. Patient decision aids were used in all studies. Standardized as well as disease-specific scales were used to measure participation (*n*=3), knowledge (*n*=3), decisional conflict (*n*=5), satisfaction (*n*=5), quality of life (*n*=2), and treatment adherence (*n* = 4) (Table 1). No studies involving critically ill children were found. The studies by Lawson, Bejarano, and Shirley reported both child-only and combined child and parent outcomes. Two studies used the same educational and treatment negotiation SDM toolkit.

**Table 1:** Study characteristics

Author Country Year Condition	Participants Age	Study design	Interventions	Outcomes
Shirley-Bejarano USA 2015 Neuromuscular scoliosis	11 children Age 8–17 yrs.	One pretest-posttest for parents knowledge about treatment characteristics	DA to help increase knowledge and satisfaction and to help decrease decisional conflict	Child and parental knowledge about treatment and the child’s condition; satisfaction with SDM tool; decision quality
Bejarano-Fuzzell USA 2015 Neuromuscular scoliosis and allergen immunotherapy	26 children and their families. Age 5–17 yrs.	Pretest.posttest, control group	DA to help increase patients/parents knowledge and satisfaction and to help increase decisional quality. To assess clinicians satisfaction with the DA To help increase treatment adherence amongst AI patients	Child and parental knowledge about treatment and provided materials; satisfaction with SDM tool; decision conflict; clinicians satisfaction with SDM
Simmons-Elmes Australia 2016 Youth depression	66 children and young adults. Age 12–25 yrs.	Pretest and posttest and follow-ups with a control group	Online DA with evidence communication, preference elicitation, and decision support components	Ability to make a decision; treatment choice; decisional conflict; satisfaction with decision (clients and clinicians); perceived involvement in decision-making
Simmons-Batchelor Australia 2017 Youth depression	229 adolescents and young adults Age 16–25 yrs.	One group pretest-posttest combined with a historical control group pretest-posttest	Online DA that peer workers help promote amongst patients.	Perceived SDM; decisional conflict; satisfaction
Liu USA 2018 Pediatric asthma	746 children Age 2–17 yrs.	Prospective cohort design with a control group	Evidence-based SDM toolkit designed for children with asthma and poor health outcomes to measure time between SDM and exacerbation	Use of SDM toolkit led to a delay in asthma exacerbation; results also showed lower probability of exacerbation
El Miedany Egypt 2019 Juvenile idiopathic arthritis	189 children Age < 16 yrs Intervention group *N* = 94 Control group *N* = 95	Posttest only, control group	Visually supported DA that informs children about treatment options, targets, side effects, and medication use	Adherence to therapy; ability to enhance clinical response; perceived involvement; treatment outcomes; school absenteeism; quality of life
Moore USA 2019 Severe obesity	31 children Age 12–17 yrs.	One group, posttest only	Patient/parent DA to support SDM between HCP and adolescents/family about two major treatment options: intensive lifestyle management and bariatric surgery pus lifestyle	Amount of SDM; self-reported knowledge; understanding risks/benefits of treatment options; value clarification; decisional conflict; self-efficacy in choosing option
Lawson Canada 2020 Type 1 diabetes	45 youths 66 parents	One group, pretest posttest	Decision coaching using a DA that empowers children to speak first in order to minimize power imbalances and to discourage parents’ interference in children’s responses	Decisional conflict (T3); choice predisposition (T2); satisfaction with decision coaching tool
Taylor USA 2018 Pediatric asthma	664 children Age 2–17 years	Pretest and posttest and follow-ups with a control group	Online and paper version of decision aid and SDM toolkit designed to facilitate three age groups: 2–4/5–11 and > 12 years.	HR QoL scores and asthma control scores

### Risk of bias assessment

**Fig. 2:**
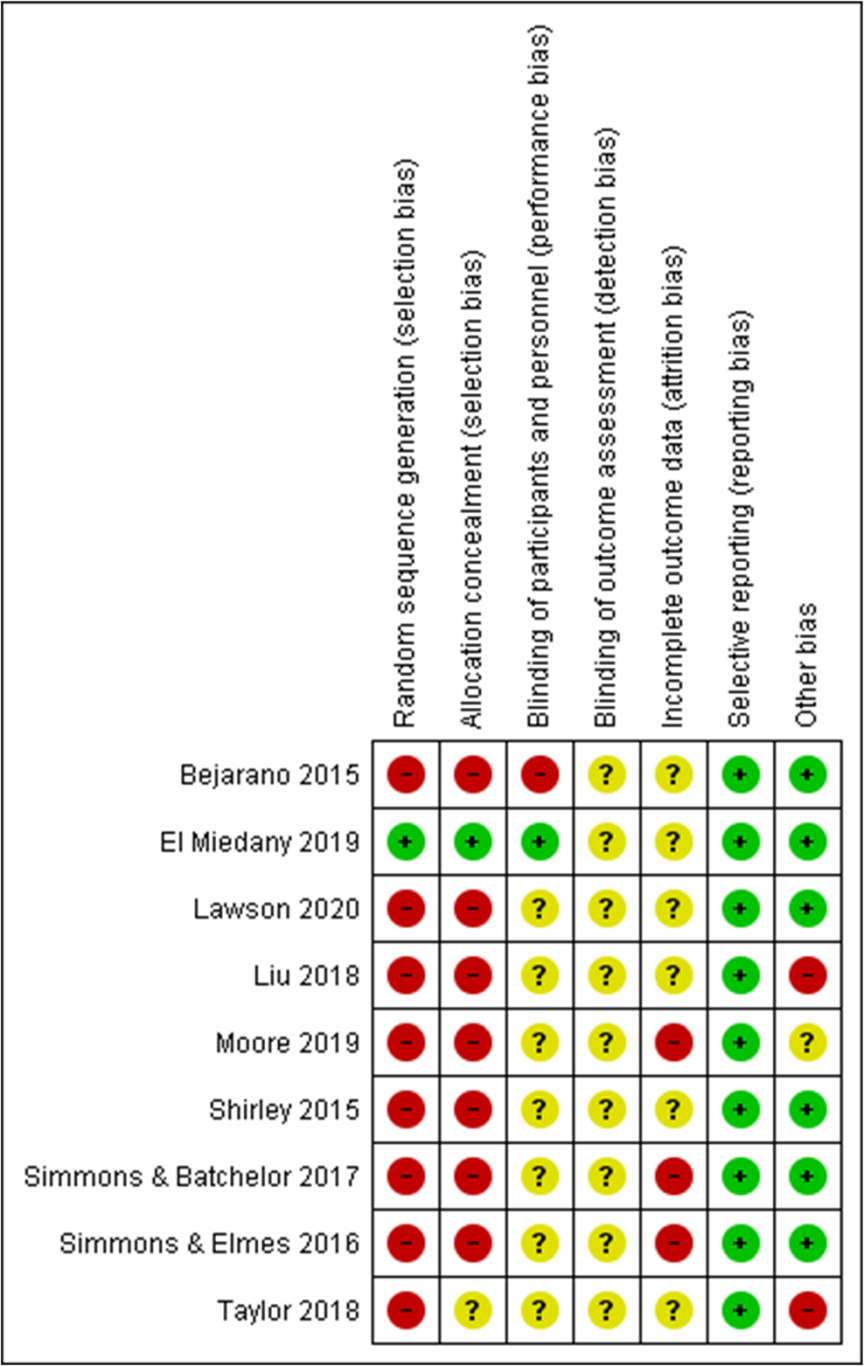
Risk of bias summary: review authors’ judgment about each risk of bias item for each included study

**Fig. 3:**
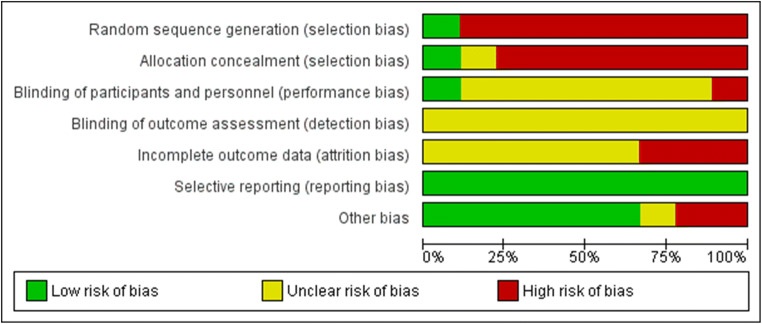
RoB overall scores. Risk of bias representing review authors’ judgments about each Rob item presented as percentages across all included studies

The authors showed considerable effort to minimize bias in their studies. Overall risk of bias was low to medium, as summarized in Figs. 2 and 3. Participants and healthcare professionals were blinded to the intervention in 11% of the studies. Risk of performance bias was low in 77% of the studies, while detection bias was unclear across all studies. One third of the studies appeared to suffer from a high risk of attrition bias, while a low risk of reporting bias was found across all studies. Only one of the eligible studies used randomization and allocation concealment.

### Results per health outcome: participation, knowledge, satisfaction, decisional conflict, health-related quality of life, and treatment adherence

#### Participation in the decision-making process

Mental health patients in Simmons-Elmes’ [25] study felt more involved in decision-making after using the decision aid, based on an 11-item SDM Questionnaire, using on a 4-point Likert scale, with scores ranging between 11 and 44. In their study, participation scores ranged from 29 to 44 (mean=37.4, SD=4.30). Likewise, Simmons-Batchelor [26] found a mean participation score of 5.46 (SD=0.59) in the intervention group of youth mental health patients, compared to 4.13 in the control group (SD=0.83, *p*<0.001). Results from the study by El Miedany [27] in juvenile idiopathic arthritis patients showed a similar pattern on three aspects of patient involvement. First, 70% of the intervention group reported being more involved by the clinician compared to 30% of the control group (RD=40%, *p*<0.01). Second, 88% of the intervention group indicated they were involved in the treatment decision-making process vs. 38% of the control group (RD=50%, no precision measure stated). Third, 89% of the intervention group reported that they reached an agreement together with their clinician about how to proceed with treatment compared to 41% of the control group (RD=48%, *p*<0.01). Overall results from Simmons-Elmes and Simmons-Batchelor indicate that SDM helped increase the level of participation in treatment decision-making [25, 26]. Results from El Miedany also indicated that SDM helped increase the level of participation regarding how to proceed with that particular treatment [27] (Fig. 5).

#### Knowledge

Simmons-Elmes showed that use of their SDM toolkit made children suffering from mental health problems more knowledgeable about the disease, which facilitated their decision-making (97% vs. 79%; *p*=0.022) compared to clinical encounters without use of the decision aid, resulting in improved adherence to depression treatment (93% vs. 70% without the decision aid; *p*=0.004).

Moore et al. [28] measured knowledge and perception of being included in the decision-making amongst children suffering from childhood obesity on multiple levels using a 13-item survey that incorporated questions from a validated 3-item CollaboRATE tool. The majority of the patients/families reported that their preferences were taken into account (8.6–8.8, range 0–9, SD=0.4). Additionally, 93% of patients/families reported better knowledge of risks and benefits of the treatment options after using the decision aid. Summarizing use of a decision aid in the youth mental health, child obesity, and neuromuscular scoliosis studies led to better knowledge of the diseases and the risk and benefits of the treatment options [25, 28, 29]. Only Bejarano reported significantly improved parent knowledge [30] (Fig. 5).

#### Decisional conflict

**Fig. 4:**
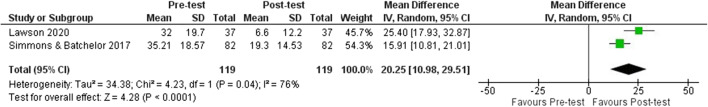
Pre- and posttest differences as a result from decisional conflict scores

**Fig. 5:**
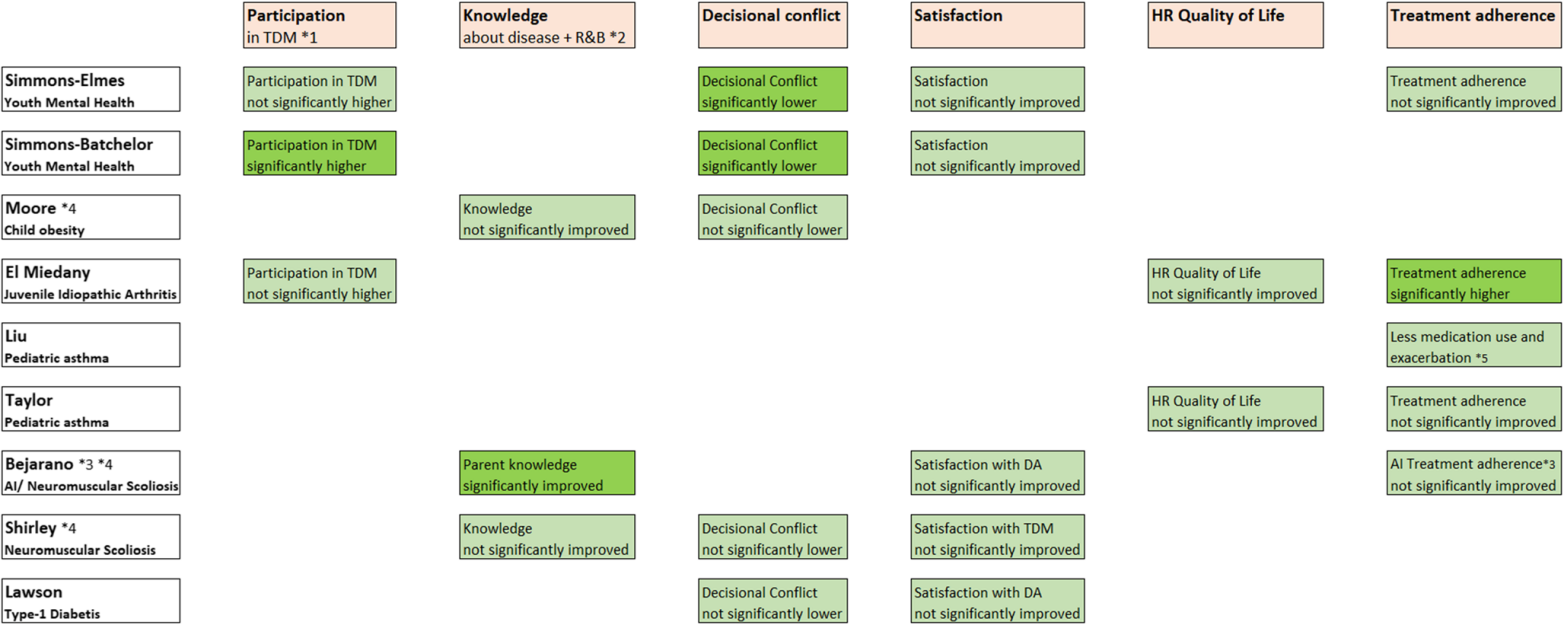
Statistical significance of health outcomes after the use of decision aids in pediatric SDM. *1 TDM, treatment decision-making. *2 R&B, risks and benefits of treatment options.*3 AI, allergenic immunotherapy. *4 Combined child and parent scores.*5 Delayed time to exacerbation and decreased risk of exacerbation

Results from Simmons-Elmes, Simmons-Batchelor, Shirley [29], and Moore and all indicated that the level of decisional conflict decreased, as a result of better knowledge about respectively youth depression, neuromuscular scoliosis, and childhood obesity treatment options and their risks and benefits. Simmons-Batchelor found that the decisional conflict post-assessment scores on the Decisional Conflict Scale (range 0–100; higher scores indicate more decisional conflict) were lower (mean=19.3, SD=14.5) than the pre-assessment scores (mean=35.2, SD=18.6) in the intervention group (see Fig. 4). The control group showed lower post-assessment scores (mean=22.0, SD=15.5) compared to the pre-assessment scores (mean=41.0, SD=16.6). Simmons-Elmes scores reported a mean decrease of 17.8 points (95%CI 13.3–22.9) in DCS scores (from 37.9 to 21.1) after using the DA 95%CI. Lawson [13] indicated that decisional conflict decreased for both parents (pretest mean 37.6, SD=20.7, posttest mean 3.5, SD=7.4, *p*<0.001) and youth (pretest mean 32, SD=19.7, posttest mean 6.6, SD=12.2, *p*<0.001) after use of the DA. Thus, Lawson’s study showed a small difference in decisional conflict between parents and youth (Fig. 4). Overall results indicated that decreased decisional conflict was related to better knowledge about the disease as well as knowledge about the risks and benefits of the different treatment options through SDM [20, 25, 26, 28, 29] (Fig. 5).

#### Satisfaction

Results from Simmons-Batchelor revealed that satisfaction was associated with perceived involvement and lower decisional conflict in the intervention and control groups of young mental health patients. However, a multiple regression model investigating the factors associated with satisfaction revealed that patient satisfaction was associated with increasing SDM participation scores (*b*=0.333, *p*=0.006) and lower decisional conflict scores (*b*= −0.295, *p*=0.013). Regarding the decision that was made, satisfaction scores were overall positive in the studies from Simmons-Elmes (youth mental health) and Shirley and Bejarano (neuromuscular scoliosis). Simmons-Elmes‘ patients expressed their satisfaction on a 6-item, 5-point Likert scale, ranging from 6 to 30. Their mean score was 25.8, SD=3.14 (95%CI 24.7–27.1). Amongst children suffering from childhood obesity Lawson reported satisfaction with the DA to help them make a balanced choice to choose a preferred option (youth 92%). Apart from children’s scores, clinicians scored a mean of 25.3 (95%CI 23.9–26.8), indicating both patients and clinicians were more satisfied with their decision after using the DA. Bejarano’s study showed a similar pattern, presenting high satisfaction scores with the intervention for both parents (mean=3.47, SD=0.78) and clinicians (mean=4.56). Both scores were measured by the 10-item parent version with a range from 0 to 4 of the Satisfaction Scale and a 5-item clinician version with a range from 1 to 5. Children’s satisfaction scores were not measured. Lawson measured satisfaction with the DA to help make a balanced choice (youth 92%, parents 96%) and, in relation to its helpfulness, to choose a preferred option (youth 92%, parents 91%). Both youth’s and parents’ scores indicated more satisfaction with the intervention. On the whole results from Simmons-Elmes, Shirley, and Bejarano indicated that satisfaction amongst respectively youth, parents, and clinicians was increased through the use of SDM [25, 29, 30] while results from Simmons-Batchelor and Lawson showed that a decreased level of decisional conflict after using the decision aid, also positively impacted Satisfaction scores [20, 26] (Fig. 5).

#### Health-related quality of life

Taylor found that use of the SDM toolkit led to increased quality of life of children suffering from pediatric asthma as measured by the MPAQL (mean difference [MD] 0.9; 95%CI 0.4–1.4) and significantly fewer asthma control problems (MD −0.9; 95%CI −1.6 to −0.2) compared to standard care. Apart from better knowledge of the disease and treatment options, use of the SDM tool also led to better health-related quality of life as measured by the Mini Pediatric Asthma Quality of Life Questionnaire (MPAQLQ). El Miedany measured quality of life of youth suffering from juvenile idiopathic arthritis in general. Also, in the light of functional disability and school absenteeism, HRQOL as measured with the c-PROMS questionnaire was also low for both groups, ranging from range 0 to 3 mean difference 0.5 (SD=0.3, 95%CI 0.44–0.56) in the intervention group to *M* = 0.9 (SD = 0.3, 95%CI 0.87–0.93) in the control group. Functional disability scores in the intervention group were mean difference 0.4 ± 0.3 (95%CI 0.34–0.46), compared to 0.8 ± 0.2 (95%CI 0.76–0.84) in the control group. School absenteeism scores were significantly better (RD=10.7%): 23% in the intervention group (95%CI 13.2–29.4) vs. 33.7% in the control group (95%CI 24.3–43.1). Overall SDM improved children’s quality of life through better disease control or, based on the results by El Miedany, improved functional disability and reduced school absenteeism [27] (Fig. 5).

#### Treatment adherence

In the study by El Miedany et al., juvenile idiopathic arthritis treatment adherence increased with the use of SDM. Similarly, Taylor reported fewer problems with asthma control after using the SDM tool compared with patients that received standard care. Bejarano’s combined child and parent scores [30] reported modest, if any improvement in treatment adherence amongst the allergenic immunotherapy population. Liu indicated that use of the SDM toolkit amongst children suffering from pediatric asthma led to less medication and lower risk of exacerbation and delayed exacerbation. Overall, patients from Taylor, Simmons-Elmes, and El Miedany reported better treatment adherence and less complications after SDM. [22, 25, 27] (Fig. 5).

## Discussion

Our scoping review shows growing awareness amongst clinicians and parents that a child’s participation in SDM can lead to better health outcomes. Previous studies show that tools to engage people in SDM are available for parents rather than for children themselves. In line with these previous studies, our results also show that more knowledge is needed about a child’s own participation and communication abilities, needs, and preferences, particularly in chronic disorders [31–33] This goes especially for children who, because of their ethnicity or demographic characteristics, are disproportionally at risk of being affected by chronic diseases such as type 1 diabetes, juvenile idiopathic arthritis, asthma, or sickle cell disease. It makes them more vulnerable to poor health outcomes. And this vulnerability also applies to the effectiveness of SDM. Studies show that apart from cognitive abilities and health literacy a child’s cultural and socioeconomic status may impact the outcome of SDM interventions [34–37]. As a result more knowledge is also needed about barriers and facilitators in pediatric SDM, especially in regard to chronic diseases such as pediatric asthma, type 1 diabetes, and child obesity that disproportionally affect children as a result of the sociodemographic and/or ethnic group that they belong to. The gaps in knowledge that we detected lead us to believe that more research is needed to determine how decision aids could help facilitate the quality of pediatric SDM, without excluding socially marginalized or otherwise disadvantaged groups. Furthermore, our study shows that more and better participation in SDM can lead to more knowledge about the disease and treatment options [32].

Better knowledge decreases decisional conflict while positively impacting treatment adherence. In line with the results from previous studies, our study also shows that treatment adherence can have a positive effect on health outcomes, such as satisfaction with the process of treatment decision-making and the chosen treatment. Increased satisfaction may positively impact a child’s health-related quality of life. Since a child’s right to participate, the right to be heard, and the right to be informed are three interdependent values, consequently, child-friendly, tailor-made, and high-quality information exchange is a prerequisite for effective and meaningful participation in SDM, one that should be part of standard procedures in clinical practice.

Overall, our study shows that several health outcomes positively impact one another through the use of pediatric SDM in a way that is comparable with adult populations. As far as critically ill children are concerned, being temporarily incapacitated to participate in shared decision-making could help clarify the lack of SDM studies for this particular group of patients. The essence of timely applying SDM techniques for children that are at risk to become critically ill is evident, either within a wider set of advance care planning measures or as part of clinical routine. Advanced care planning programs could be useful to promote SDM when dealing with critically ill children and their families [38, 39]. Given the rapid developments in medical technology, more research is needed to explore how technologically advanced decision aids could help facilitate the quality of pediatric SDM, without excluding socially marginalized or otherwise disadvantaged groups. Furthermore, the design of SDM scales and tools that—apart from internal reliability—are also specifically validated for pediatric SDM should be encouraged [40, 41]. And finally, given the importance of the information exchange that belies SDM, more knowledge is needed how to incorporate the pivotal role of the nursing staff within pediatric SDM [42]. Several family-integrated care programs show promising results with this role of the nursing staff as a specialized family consultant [43]. In summary, the desire is growing for the promotion of SDM in pediatrics, not only by children and their parents but also by pediatricians [28]. This desire may be accommodated by adding recommendations in clinical practice guidelines towards SDM and both analog and digital tools for this purpose [6]. At the same time, more awareness amongst healthcare professionals, parents, and policy makers is needed about the multidimensional and complex character and key features of pediatric SDM compared to SDM with adults. In their best interest, chronically ill children’s information needs have to be addressed first [44–49],. Such based on their cognitive abilities, as well as their communication abilities, needs, and preferences [50, 51]. This holds true especially for socially vulnerable children that are at greater risk for being affected by chronic diseases.

### Study limitations

Some limitations of our review need to be mentioned. One limitation is the small number of studies we found that fitted our eligibility criteria. Nevertheless, this review shows the best available evidence about this important subject. Second, none of the included studies involved critically ill children as a target audience. Apparently, these circumstances seem less inviting to engage these children and their parents in SDM; however, the decisions that need to be made are important.

A third limitation is the lack of randomized trials, making causal relations harder to prove. Finally, some studies consisted of small populations, while others used scales that have been validated and commonly used among adult patients, but were not specifically designed for use in the realm of pediatrics. Therefore, our results need to be read with a certain amount of caution. Nevertheless, we believe these drawbacks do not substantially change our main findings and conclusions.

## Conclusion

To minimize health disparities, a child-friendly and high-quality information exchange with the child is a prerequisite for their effective and meaningful participation in SDM. Barriers and facilitators in pediatric SDM need to be addressed, especially concerning chronic diseases such as pediatric asthma, type 1 diabetes, and child obesity that disproportionally affect children as a result of the sociodemographic and/or ethnic group that they belong to. This makes these children more vulnerable to poor health outcomes. And this social vulnerability could accumulate to insufficient effectiveness of SDM techniques unless more evidence becomes available:

- How advanced, age-appropriate, and child-friendly technological decision aids can help facilitate the quality of pediatric SDM without excluding socially marginalized children,
- which SDM scales apart from internal reliability are specifically validated for pediatric SDM, and
- how to incorporate the pivotal role of the nursing staff within pediatric SDM.

Closing these knowledge gaps may be fostered by the surging mobile, virtual, and augmented health technologies. It is in the child’s best interest that gaps in knowledge about a child’s participation preferences in SDM are addressed, only then can we fully get insight in a chronically ill child’s participation abilities, needs, and preferences as a prerequisite for full-fledged and mature pediatric SDM.

## Data Availability

All data generated or analyzed during this study are included in this published article and its supplementary information files.

## Appendix: Search strategy

**Table:**

**#**	**Search**	**Results**
	**Ovid MEDLINE(R) ALL** **Search date: 17 January 2020**	
1	exp child/ or infant/ or exp child welfare/ or exp puberty	2118968
2	(school age or adolescen* or teen age? or teens or youth? or puberty or prepubescen* or infant? or infancy or toddler? or kid or kids or underage* or boy or boys or girl? or sibbling* or preschool* or childhood or child or children or schoolchild* or juvenile or minors or p?ediatric?).ab,kf,ti.	2099724
3	((“2” or “3” or “4” or “5” or “6” or “7” or “8” or “9” or “10” or “11” or “12” or “13” or “14” or “15” or “16” or “17” or “18”) adj1 (age? or yr? or year?)).ab.	1406500
4	(adolescen* or child or p?ediatric? or juvenile).jw.	572205
5	or/1–4 [children 2–18 yrs]	3898124
6	critical illness/	27561
7	(critical* adj2 (condition? or disease? or disorder? or ill*)).ab,kf,ti.	55613
8	exp chronic disease/	260597
9	((chronic* adj2 ill*) or chronic disease? or chronic disorder? or chronic condition? or noncommunicable or “non-communicable”).ab,kf,ti.	110691
10	exp terminal care/ or “hospice and palliative care nursing”/ or hospices/ or palliative care/ or palliative medicine/	95743
11	(eol or “end of life” or advance care or hospice? or resuscitation or euthanasia or assisted suicide? or palliative or terminal care).ab,kf,ti.	158626
12	or/6–11 [chronically or critically ill]	594527
13	5 and 12 [target population]	115760
14	((shared decision adj2 making) or sdm).ab,kf,ti.	9115
15	((clinical decision? or medical decision? or decision making) adj4 (involv* or participat* or engage* or (child* adj2 perspective?) or triad* or dyad*)).ab,hw,kf,ti.	7814
16	14 or 15 [shared decision making]	15774
17	patient participation.ab,hw,kf,ti. or stakeholder participation/	27207
18	patient centered care/ or (child centered care or patient centered care).ab,kf,ti.	21345
19	(communication and ((doctor or physician or provider) adj3 (child* or pe?diatric*))).ab,kf,ti.	197
20	((child* or pe?diatric*) adj2 (involv* or participat* or engage* or perspective? or value?)).ab,kf,ti.	23399
21	or/17–20 [communication | information]	70082
22	(5 and 16) or (13 and 21)	4187
23	limit 22 to yr=“2008–current”	3135
	**Ovid PsycINFO** **Search date: 17 January 2020**	
1	schools/ or boarding schools/ or elementary schools/ or high schools/ or junior high schools/ or kindergartens/ or middle schools/ or community colleges/ or puberty/	54884
2	(youngster or pubert* or pubescent or prepubescent or school or schools or schoolkid* or schoolchild* or highschool* or kid or kids or underage* or youth? or boy or boys or girl? or sibbling* or preschool* or toddler? or child or children or schoolchild* or adolescents or adolescence or juvenile or minors or teen or teens or teenager* or p?ediatric?).ab,id,ti.	1049422
3	(adolescen* or child or p?editric? or adolescents or adolescence or juvenile).jx.	123559
4	((“3” or “4” or “5” or “6” or “7” or “8” or “9” or “10” or “11” or “12” or “13” or “14” or “15” or “16” or “17” or “18”) adj1 (age? or yr? or year?)).ab.	364898
5	(“160” or “180” or “200”).ag.	640644
6	or/1–5 [children 2–18 yrs]	1326530
7	chronic illness/ or chronic mental illness/ or chronic pain/ or chronically ill children/ or “chronicity (disorders)”/	30250
8	(critical* adj2 (condition? or disease? or disorder? or ill*)).ab,id,ti.	3364
9	((chronic* adj2 ill*) or chronic disease? or chronic disorder? or chronic condition? or noncommunicable or “non-communicable”).ab,id,ti.	31558
10	terminally ill patients/ or palliative care/ or hospice/	16298
11	(eol or “end of life” or advance care or hospice? or resuscitation or euthanasia or assisted suicide? or palliative or terminal care).ab,id,ti.	23002
12	or/7–11 [chronically or critically ill]	77730
13	6 and 12 [target population]	17052
14	((shared decision adj2 making) or sdm).ab,id,ti.	2796
15	((clinical decision? or medical decision? or decision making) adj4 (involv* or participat* or engage* or (child* adj2 perspective?) or triad* or dyad*)).ab,id,ti.	6578
16	14 or 15 [shared decision making]	8880
17	communication/	25624
18	communication.ab,id,ti.	169582
19	(client participation or patient participation).ab,id,ti. or client participation/	2912
20	client centered therapy/ or (child centered care or patient centered care).ab,id,ti.	4525
21	((child* or pe?diatric*) adj2 (involv* or participat* or engage* or perspective? or value?)).ab,id,ti.	22947
22	or/17–21 [communication | information]	201722
23	(6 and 16) or (13 and 22)	3612
24	limit 23 to yr=“2008–current”	2332
	**Ebscohost: Communication & Mass Media Complete** **Search date: 17 January 2020**	
S16	S14 OR S15	858
S15	S3 AND S12 limit: 2008–current	837
S14	S7 AND S12	56
S13	S3 AND S12	1234
S12	S8 OR S9 OR S10 OR S11	1819
S11	AB ((child* or pe?diatric*) N1 (involv* or participat* or engage* or perspective? or value?)) OR KW ((child* or pe?diatric*) N1 (involv* or participat* or engage* or perspective? or value?)) OR SU ((child* or pe?diatric*) N1 (involv* or participat* or engage* or perspective? or value?)) OR TI ((child* or pe?diatric*) N1 (involv* or participat* or engage* or perspective? or value?))	901
S10	AB client participation or patient participation or child centered care or patient centered care OR KW client participation or patient participation or child centered care or patient centered care OR SU client participation or patient participation or child centered care or patient centered care OR TI client participation or patient participation or child centered care or patient centered care	483
S9	AB ((clinical decision? or medical decision? or decision making) N3 (involv* or participat* or engage* or (child* N1 perspective?) or triad* or dyad*)) OR KW ((clinical decision? or medical decision? or decision making) N3 (involv* or participat* or engage* or (child* N1 perspective?) or triad* or dyad*)) OR SU ((clinical decision? or medical decision? or decision making) N3 (involv* or participat* or engage* or (child* N1 perspective?) or triad* or dyad*)) OR TI ((clinical decision? or medical decision? or decision making) N3 (involv* or participat* or engage* or (child* N1 perspective?) or triad* or dyad*))	372
S8	AB ((shared decision N1 making) or sdm) OR KW ((shared decision N1 making) or sdm) OR SU ((shared decision N1 making) or sdm) OR TI ((shared decision N1 making) or sdm)	146
S7	S4 OR S5 OR S6	2301
S6	AB eol or “end of life” or advance care or hospice? or resuscitation or euthanasia or assisted suicide? or palliative or terminal care OR KW eol or “end of life” or advance care or hospice? or resuscitation or euthanasia or assisted suicide? or palliative or terminal care OR SU eol or “end of life” or advance care or hospice? or resuscitation or euthanasia or assisted suicide? or palliative or terminal care OR TI eol or “end of life” or advance care or hospice? or resuscitation or euthanasia or assisted suicide? or palliative or terminal care	1725
S5	AB ((chronic* N1 ill*) or chronic disease? or chronic disorder? or chronic condition? or noncommunicable or “non-communicable”) OR KW ((chronic* N1 ill*) or chronic disease? or chronic disorder? or chronic condition? or noncommunicable or “non-communicable”) OR SU ((chronic* N1 ill*) or chronic disease? or chronic disorder? or chronic condition? or noncommunicable or “non-communicable”) OR TI ((chronic* N1 ill*) or chronic disease? or chronic disorder? or chronic condition? or noncommunicable or “non-communicable”)	468
S4	AB (critical* N1 (condition? or disease? or disorder? or ill*)) OR KW (critical* N1 (condition? or disease? or disorder? or ill*)) OR SU (critical* N1 (condition? or disease? or disorder? or ill*)) OR TI (critical* N1 (condition? or disease? or disorder? or ill*))	119
S3	S1 OR S2	130541
S2	SO adolescen* or child or p?editric? or adolescents or adolescence or juvenile	42201
S1	AB youngster or pubert* or pubescent or prepubescent or school or schools or schoolkid* or schoolchild* or highschool* or kid or kids or underage* or youth? or boy or boys or girl? or sibbling* or preschool* or toddler? or child or children or schoolchild* or adolescents or adolescence or juvenile or minors or teen or teens or teenager* or p?ediatric? OR KW youngster or pubert* or pubescent or prepubescent or school or schools or schoolkid* or schoolchild* or highschool* or kid or kids or underage* or youth? or boy or boys or girl? or sibbling* or preschool* or toddler? or child or children or schoolchild* or adolescents or adolescence or juvenile or minors or teen or teens or teenager* or p?ediatric? OR SU youngster or pubert* or pubescent or prepubescent or school or schools or schoolkid* or schoolchild* or highschool* or kid or kids or underage* or youth? or boy or boys or girl? or sibbling* or preschool* or toddler? or child or children or schoolchild* or adolescents or adolescence or juvenile or minors or teen or teens or teenager* or p?ediatric? OR TI youngster or pubert* or pubescent or prepubescent or school or schools or schoolkid* or schoolchild* or highschool* or kid or kids or underage* or youth? or boy or boys or girl? or sibbling* or preschool* or toddler? or child or children or schoolchild* or adolescents or adolescence or juvenile or minors or teen or teens or teenager* or p?ediatric?	130541
	**Web of Science Core Collection** **Search date: 17 January 2020**	
#18	(#3 and #13 and #17) or (#8 and #13)	2277
#17	#14 OR #15 OR #16	213496
#16	TS=((child* or pe?diatric*) NEAR/1 (involv* or participat* or engage* or perspective? or value?))	23406
#15	TS=(communication and (doctor or physician or provider NEAR/2 (child* or pe?diatric*)))	28575
#14	TS=(patient participation or child centered care or patient centered care)	167985
#13	#9 OR #10 OR #11 OR #12	50838
#12	TS=(decision making NEAR/3 (involv* or participat* or engage* or triad* or dyad*))	36887
#11	TS=(medical decision? NEAR/3 (involv* or participat* or engage* or triad* or dyad*))	3451
#10	TS=(clinical decision? NEAR/3 (involv* or participat* or engage* or triad* or dyad*))	4724
#9	TS=((shared decision NEAR/1 making) or sdm)	12581
#8	#3 AND #7	105573
#7	#4 OR #5 OR #6	565357
#6	TS=(eol or “end of life” or advance care or hospice? or resuscitation or euthanasia or assisted suicide? or palliative or terminal care)	216282
#5	TS=((chronic* NEAR/1 ill*) or chronic disease? or chronic disorder? or chronic condition? or noncommunicable or “non-communicable”)	284790
#4	TS=(critical* NEAR/1 (condition? or disease? or disorder? or ill*))	77868
#3	#1 OR #2	3424699
#2	AB=((“2” or “3” or “4” or “5” or “6” or “7” or “8” or “9” or “10” or “11” or “12” or “13” or “14” or “15” or “16” or “17” or “18”) NEAR/1 (age? or yr? or year?))	1277897
#1	TS=(school age or adolescen* or teen age? or teens or youth? or puberty or prepubescen* or infant? or infancy or toddler? or kid or kids or underage* or boy or boys or girl? or sibbling* or preschool* or childhood or child or children or schoolchild* or juvenile or minors or p?ediatric?)	2556614

## Abbreviations

DA: Decision aid
HR QoL: Health-related quality of life
JIA: Juvenile inflammatory arthritis
MPAQLQ: Mini Pediatric Asthma Quality of Life Questionnaire
RoB: Risk of bias
SDM: Shared decision-making
TDM: Treatment decision-making
